# Sun Protection Counseling at the Pharmacy: A German Qualitative Study on Status Quo, Potential Deficits, and Sources of Information

**DOI:** 10.3390/healthcare11131907

**Published:** 2023-06-30

**Authors:** Katharina Diehl, Maike Carola Battenberg, Charlotte Jansen, Tatiana Görig

**Affiliations:** 1 Department of Medical Informatics, Biometry and Epidemiology, Professorship of Epidemiology and Public Health, Friedrich-Alexander-Universität Erlangen-Nürnberg (FAU), 91054 Erlangen, Germany; 2 Medical Faculty Mannheim, Heidelberg University, 68167 Mannheim, Germany

**Keywords:** skin cancer, pharmacy, sunscreen, sun protection

## Abstract

The sale of sunscreen products is lucrative for pharmacies, and many people buy sunscreen in pharmacies because they expect to receive good advice on sunscreen use and sun protection. However, little is known about the knowledge level of pharmacists and pharmacy technicians in the field of skin cancer prevention. By conducting a qualitative study in Germany, we aimed to explore what pharmacy personnel know about sun protection and the proper use of sunscreens, where they get their information from, and where they see deficits concerning these topics. We found that there is a need for education regarding the terms skin type and sun protection factor, both of which can be helpful tools when advising customers on sun protection. In addition, participants wished for more independent information from health authorities because sunscreen manufacturing companies, which offer product-specific information, are often the only source of information. Overall, it seems reasonable that pharmacy staff should be educated more about sun protection behavior and the proper use of sunscreen to be able to advise their customers correctly. Our findings offer a starting point for strengthening the role of pharmacies in skin cancer prevention. It seems to make sense to develop and offer tailored handouts for customer counseling. Since sunscreen products are perceived as seasonal products, an educational leaflet can help refresh knowledge about the use of sunscreen and the concepts of skin type and sun protection factor in early summer.

## 1. Introduction

The incidence of skin cancer has increased in Western industrialized countries in recent decades [1]. This applies to malignant melanoma as well as keratinocyte carcinoma, which is the most common cancer type and comprises basal cell carcinomas and squamous cell carcinomas [2]. Current data from the World Health Organization (WHO) revealed that one in every three diagnosed cancers is skin cancer [2]. 

Ultraviolet radiation is the most important risk factor for the development of skin cancer. Therefore, natural sun exposure and tanning bed use have been classified as carcinogenic to humans by the International Agency for Research on Cancer [3]. Besides avoiding ultraviolet radiation, sun protection is a key measure for skin cancer prevention.

In addition to seeking shade, wearing sun-protective clothes, hats, and sunglasses, the use of sunscreens is an important measure to protect from the sun [4,5]. Representative data from Germany showed that 43.8% of the general population aged 14 to 45 years used sunscreen always or often on sunny summer days [6]. Worldwide, 6.9 billion EUR were spent on sunscreen in 2021 [7]. In Germany, the expenditure on sunscreen products amounted to 168.5 million EUR in 2021 [8]. As in many other countries, sunscreens can be purchased in supermarkets, drugstores, and pharmacies in Germany [9].

In 2020, pharmacies in Germany generated approximately 96 million EUR of gross sales from the sale of sunscreen [10]. The most important reason for purchasing sunscreen in pharmacies is the desire for expert advice [11,12]. International data show that pharmacists are considered as credible and trustworthy [13,14]. According to Tucker and Duffy [15], counseling on sun safety is an important activity for health promotion in pharmacies.

In German pharmacies, two professional groups are typically represented: pharmacy technicians and pharmacists. In Germany, pharmacy technicians are trained in state-recognized vocational schools. The training lasts 2.5 years and includes theoretical training (2 years) and a six-month internship. Theoretical, practical, and general education subjects are taught [16]. These include, among others, pharmacology, chemistry, medicine production, botany, nutritional science, and body care science [16]. The latter includes, for instance, skin and hair care as well as sun protection, and is a non-examination subject [17]. The tasks of pharmacy technicians include, amongst others, counseling customers (including providing information about products such as cosmetics or sunscreen products), dispensing medications, manufacturing medications (e.g., ointments) under the supervision of pharmacists, performing services such as measuring blood pressure, and administrative tasks. In Germany, pharmacists complete a university course of study within a standard period of eight semesters, followed by a state examination. After study or training, both occupational groups can be offered in-service training by various providers [18]. These can include specific training courses on sun protection. In Canada, for example, sun protection is typically taught in courses called “self-care”. One study found that 90% of Canadian pharmacy school curricula include lessons on sunburn and sunscreen [19].

As previous research and surveys have shown that pharmacies are an important and trusted point of contact for the sale of and advice on sunscreen and its proper use, our study aimed to explore the situation of sale and counseling in pharmacies by gathering information from pharmacists and pharmacy technicians. We focused on the subjectively perceived importance of counseling on sun protection in German pharmacies and the knowledge of pharmacy staff on sun protection and sunscreen, as well as counseling on sun protection measures in their daily business. The findings will help to understand how counseling is performed and where pharmacists and pharmacy technicians see deficits and wish for support if necessary.

## 2. Materials and Methods

We conducted a qualitative study consisting of semi-structured interviews with 20 pharmacists and pharmacy technicians in Germany [9]. The Ethics Committee II of the Medical Faculty Mannheim, Heidelberg University, approved the study on 26 February 2019 (number: 2019-1154N). Before the interviews, all participants were informed about the study procedure and data protection. All participants provided written consent to participate in the study. They received a 20 EUR gift voucher as compensation for their time.

### 2.1. Recruitment of Participants

The participants were recruited from the Rhine-Neckar region in southwestern Germany through pharmacies located in urban and rural areas [9]. Addresses of pharmacies in this region were obtained from the yellow pages directory. Pharmacies were contacted through letters of invitation and direct contact at the pharmacy. Pharmacists and pharmacy technicians who were able to participate in the interview in German were eligible to participate. No other inclusion or exclusion criteria were applied. We included new participants in the study until we felt that the information gained from additional interviews decreased with an increasing number of participants. This means that additional interviews did not reveal any new information beyond the aspects already identified (theoretical saturation). Our saturation point was reached at approximately interview 17, and we, therefore, stopped data collection after 20 interviews.

### 2.2. Data Collection

We used a semi-structured guide with open-ended questions focusing on different themes. This manuscript addresses the subjectively perceived importance of sun protection, pharmacists’ and pharmacy technicians’ knowledge of sun protection, and counseling on sun protection measures in the pharmacy. In addition, three case studies on counseling situations were discussed:

- Case study I: “I would like you to imagine that a customer comes to the pharmacy where you work at the beginning of May and wants to be advised by you. He wants to take a summer vacation in Mallorca with his children and his wife and asks you what he should pay special attention to in terms of sun protection.”
- Case study II: “Please imagine that a young mother enters the pharmacy with her infant. She wants to spend her summer vacation in the south of Spain and asks you about sun protection for her child. What do you recommend to her?”
- Case study III: “Imagine an elderly lady comes up to you with a bottle of sunscreen that she has taken from the display and wants to know what the term “sun protection factor” means. What would you say? The lady takes several medications and is asking if she needs to take special care when going out in the sun.”

All interviews were conducted face to face from May to December 2019 by MCB (female medical student and graduated nurse), who was extensively trained in advance by the first author (KD), who is experienced in qualitative interviewing [9]. MCB conducted two pretest interviews to pilot the semi-structured interview guide. The practicability of the semi-structured guide was good resulting in a fluent interview process. The understandability of interview questions was perceived as good because no queries regarding questions and terms used occurred. Therefore, the pretest interviews were included in the analysis. Interviews lasted 35 min on average (min: 25 min, max: 55 min) [9]. For the interviews, the participants suggested places where they felt comfortable talking. No third parties were present during the interviews. All interviews were audiotaped (Olympus WS-853) and transcribed verbatim by MCB and CJ.

### 2.3. Data Analysis

We used qualitative content analysis according to Mayring [20] to analyze the data. We systemized the data by identifying categories and subcategories (i.e., common themes within the interviews). Based on an initial set of main codes based on the semi-structured interview guide, the codes were further refined during the process of coding. For the coding, we used the program MAXQDA (VERBI Software GmbH, Berlin, Germany). The interviews were independently coded by two researchers (MCB and CJ). The kappa value for consensus, following Brennan and Prediger [21], was 0.82, which indicates a high agreement level. Disagreements in coding were discussed and resolved by consensus in each case.

## 3. Results

A total of 20 pharmacists and pharmacy technicians participated in the interviews. Of these participants, 16 were female (80%), and 4 were male (20%). The mean age was 47.5 years (age range: 23 to 69 years). Half of the interviewees had completed vocational training/apprenticeship (50%) and half had a university degree (50% master’s degree (M.A.), diploma or doctorate). The date of graduation ranged from 1980 to 2019 (M: 1996; SD: 13.8). Of the 20 participants, 4 reported being the owner or manager of a pharmacy (20%), 6 others were employed pharmacists (30%), and 10 interviewees worked as pharmacy technical assistants (50%). Slightly more than half of the interviewees worked full-time (60%).

On average, the pharmacies had about twelve employees. The pharmacies were predominantly located in urban areas (75%). Three-quarters of the respondents reported that they had had further training in sun protection since the completion of their training/studies.

### 3.1. Perceived Importance of Sun Protection

About half of the respondents indicated that sun protection is very important in the pharmacy they work for. For example, P07 stated that “*the topic of sun protection is of great importance and is also requested by customers*”. P01 reported that “*it has decreased in the last few years. In the past, when I started, it was clearly more. People also wanted to have proper advice, more sunscreen products were sold. This is just now decreasing more and more*”. Seven participants reported that the importance depended on the season.

A few interviewees said that counseling on sun protection plays a rather minor role in the pharmacy where they work. Among these two is P19, who reported that the importance of sun protection was “*low. Is really low. So it’s very rarely asked for. It used to be more in the cities maybe more, but hardly ever in the countryside”* and gives a reason: *“The way I explain it is that people […] already know that you have to put on sunscreen, and I also have the feeling that the mothers know well that they have to protect their children from the sun from the very beginning, but the sunscreen is not bought in the pharmacy. It is bought in the drugstores.*”

Sun protection hardly played a role in the training or during studies of the participating pharmacists and pharmacy technicians. The majority stated that the topic had not been addressed at all (e.g., P01 “*Well, not at all during training at [vocational] school.*”). Others reported that sun protection had played a minor role in their professional training (e.g., P20 “*Marginal. Once in an hour briefly checked off.*”).

As a result of this perceived lack of knowledge, most of the participants attended training courses on sun protection or gained knowledge by reading articles. The training courses were mainly offered by sunscreen product manufacturers, but also courses organized by professional associations (e.g., State Pharmacy Association, P17) were named.

### 3.2. Knowledge of Sun Protection

#### 3.2.1. Knowledge of Sunscreen Use

Regarding proper sunscreen use, we included two different questions in our interviews. First, we asked respondents about their recommendations to customers when asked how much sunscreen customers should apply for optimal sun protection. Regarding the amount of sunscreen to use, many respondents indicated a sufficient amount, while some participants could not commit to any amount. A minority would advise the customer to use too little sunscreen (Table 1). The general recommendation for sunscreen is 2 mg of product per cm^2^ of the skin surface, which is approximately 40 g of sunscreen for a full body application for a typical adult [22].

**Figure 1:**
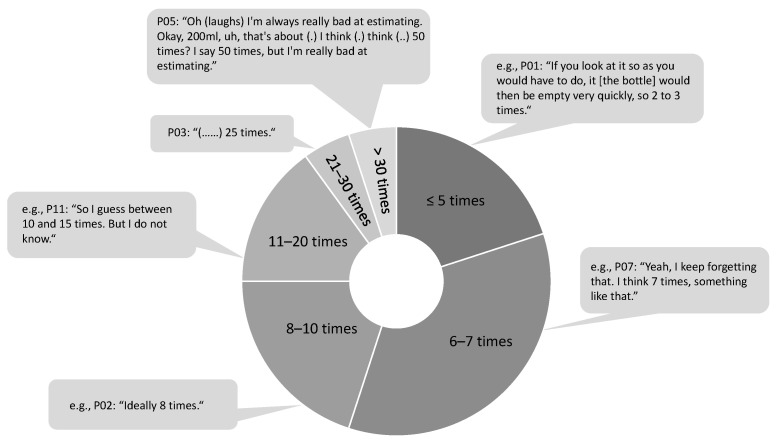
Estimated number of times an adult can apply sunscreen to their entire body using a 200 ml bottle of sunscreen.

Second, we asked the participants to think about a 200 mL bottle of sunscreen. We wanted to know how many times they thought an average-sized, average-weight adult could apply sunscreen to their entire body using this 200 mL bottle [23,24]. We found a wide range of answers (Figure 1). The correct answer would be 5 times for an average person [22,23,24]. A few participants correctly indicated that such a bottle would be sufficient for five applications, while some additional participants were only slightly above this with six to seven times.

#### 3.2.2. Sources of Information

Sources of information on sun protection included companies that manufacture and sell sunscreen (n = 18), pharmacy journals (n = 13), the internet (n = 5), training courses (n = 4), physicians (n = 2), and official guidelines (n = 1; multiple responses were possible). The majority would like to have more information on the subject of sun protection. Nearly all of these respondents expressed a desire for independent information that is not tied to product manufacturers. Examples of these comments are shown in Table 2.

#### 3.2.3. Knowledge of Skin Types

Regarding the classification of skin types according to Fitzpatrick [25], a categorization into six different types based on ultraviolet sensitivity, only a minority (P02, P10, and P14) was able to describe that there are six different types and what the classification is based on. Sixteen participants could name and describe at least partial aspects of the skin type classification, while one participant (P05) had no idea. She thought that the interviewer meant “*dry skin, combination skin, oily skin*” (P05).

Among those who were at least partially aware of the classification (n = 19), the majority of participants agreed that they could classify skin types well, while some (P01, P09, P10, P15, P16, and P19) had doubts about their ability to do so. One participant (P06) thought that it would not be easy.

When asked about the interviewer’s skin type (skin type II), six participants were able to give a correct answer (P01, P04, P07, P08, P12, and P13). Another six participants gave an incorrect answer, three of whom underestimated the skin type (P10, P15, and P17) by indicating skin type I, and three of whom overestimated the skin type (P02, P06, and P14). However, most of the latter reported skin type III, so there was no excessive overestimation. Seven participants were unable to determine the interviewer’s skin type according to Fitzpatrick’s classification [25].

**Figure 2:**
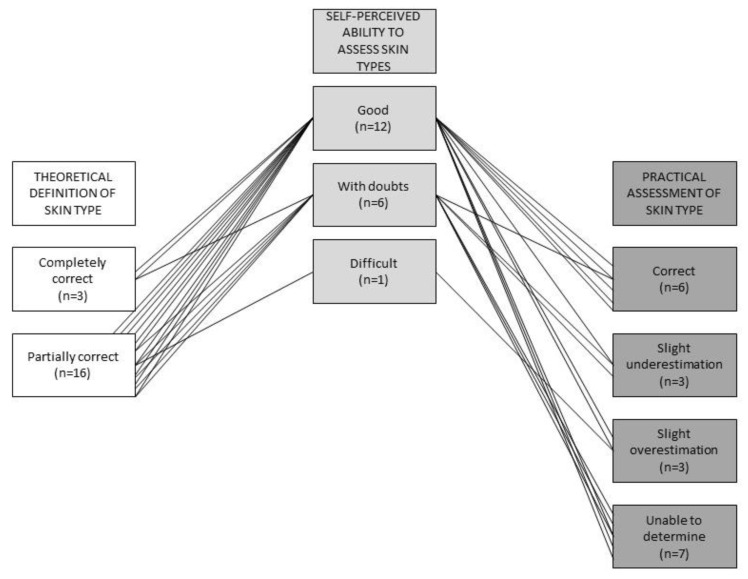
Relationship between self-perceived ability to assess skin type based on the Fitzpatrick classification with knowledge of Fitzpatrick’s definition and ability to practically assess the skin type of the interviewer. n = 19, one participant was excluded because she had never heard the term skin type before.

Figure 2 illustrates several aspects: The starting point of the figure is the self-perceived ability to assess skin types according to Fitzpatrick’s definition. Two out of twelve respondents who said that they were good at assessing skin types were able to give a correct definition of skin type according to Fitzpatrick. When asked to rate the interviewer’s skin type, five of the twelve respondents with a good self-perceived ability were able to give a correct answer, while four of them were unable to determine a skin type.

### 3.3. Counseling on Sun Protection Measures

Participants were asked what they thought were the reasons why individuals buy sunscreen in pharmacies. The answers can be summarized in four reasons: desire for personal advice, i.e., especially customers with a need for advice (e.g., skin diseases, sensitive skin, and mothers with children, n = 12) come to the pharmacy; higher (expected) product quality than in drugstores or supermarkets (n = 11); higher competency of staff than in drugstores or supermarkets (n = 5); and possible overloading of the product range in drugstores (n = 1).

The number of counseling sessions on sun protection varied widely among the participants. All of them agreed that the frequency of counseling depends on the time of year. For example, in summer and before the holidays, but also in winter before the winter vacations, customers come to ask for advice.

About a third (P03, P04, P06, P08, P09, P13, and P16) would recommend a dietary supplement such as beta-carotene or calcium to prepare the skin for sun exposure during the summer vacation, while another third (P05, P07, P10, P17, P18, and P19) would not. P01, P02, P11, P12, P14, P15, and P20 were indifferent to the recommendation of dietary supplementation to prepare the skin for ultraviolet exposure and would neither actively recommend it nor advise clients against it.

#### 3.3.1. Case Study I

The first case involved a customer who was planning a summer vacation in Mallorca with his children and wife and wanted advice on what to look out for in terms of sun protection. Ten different aspects were coded based on the content of the interviews, which were provided without detailed probing by the interviewer (Table 3). The mean number of aspects per interview was 4.3, with a minimum of 1 and a maximum of 8. None of the participants mentioned the use of sunglasses. The least frequently mentioned aspects were specific instructions for sunscreen application (e.g., reapplication), the type of sunscreen recommended (e.g., high sun protection factor), and other recommendations for sun protection behaviors beyond sunscreen use (e.g., wearing a hat).

Detailed questions about sunscreen use revealed differing opinions about when sunscreen should be applied. Several health authorities recommend applying sunscreen at least 30 min before sun exposure and reapplying it every two hours or after sweating or swimming [26,27,28,29]. Most of the participants (P02, P03, P04, P05, P06, P08, P09, and P19) recommended that their customers apply sunscreen about 15 min before leaving the house. Some participants (P10, P11, P15, P17, and P20) recommended applying sunscreen about 30 min before, while other participants (P12, P13, P14, and P18) recommended applying sunscreen immediately before leaving the house. A minority did not give a specific answer (P01, P07, and P16).

All participants agreed that individuals should reapply sunscreen. However, they had different opinions about when to reapply sunscreen. It is recommended to reapply the sunscreen every two hours or after swimming or sweating [26,27,28,29]. Some (P08 and P16) recommended reapplication after one to two hours, others (P01, P03, P09, P11, P14, P16, and P18) recommended reapplication after two to four hours, and others (P10 and P20) after more than four hours. Some other participants reported that the reapplication time depends on the skin type (e.g., P19) and is a more individual aspect. All participants except P07 felt that sunscreen should be reapplied after swimming, and about half of the participants (P01, P02, P09, P13, P14, P15, P18, P19, and P20) stressed the importance of reapplying sunscreen after sports and after sweating.

#### 3.3.2. Case Study II

The second case study focused on a mother who wanted to take her infant on a summer vacation in Spain. All participants agreed that an infant needs to be well protected. Nineteen participants recommended a combination of sun protection measures (e.g., clothing, shade, and sunscreen). One participant (P05) recommended the use of sunscreen alone. While many interviewees emphasized that an infant should not be exposed to the sun at all, P11 stated that children should be exposed to the sun for short periods, “*but not in the blazing sun, […] simply because the blood circulation may not be able to handle it either. […] It could be 10 min. […] This is also sometimes done with children who are jaundiced, that they undress them and then put them in the sun.*”

#### 3.3.3. Case Study III

Case study III involved an elderly woman who wanted to know what the term “sun protection factor” means. She also wanted to know if she needs to take any special precautions when out in the sun as she is on medication.

Most of the participants were able to explain the meaning of sun protection factor, at least in general terms. The other participants (P01, P03, P05, P08, and P11) answered the question rather vaguely or incorrectly. E.g., P11 said: “*There is now also a formula, but I honestly do not have it at hand.”* And P03 answered *“I don’t remember how it was connected. It has changed over the years, what is meant by it. But I don’t have it exactly in my head anymore. Yes, I do not know now. I would have to look again.*”

Regarding possible increased sensitivity to ultraviolet radiation due to medication use, all participants agreed that this could be a problem for the woman. She should be asked what medications she is taking, and then, it should be clarified whether these can lead to increased ultraviolet sensitivity. With the exception of P05, all others gave examples of medications that the woman should be aware of, e.g., antibiotics and St. John’s wort.

## 4. Discussion

Previous research has shown that pharmacies can be good places for skin cancer prevention [30]. However, effective prevention work requires sufficient knowledge and competence to advise customers appropriately. To date, only a few studies have focused on pharmacists’ knowledge of and counseling regarding sun protection [31]. Previous studies from Arizona (USA) and Iran have shown deficits in the knowledge of pharmacy personnel [32,33]. However, it was shown that training pharmacy personnel could increase knowledge, self-rated expertise, and the proportion of patients counseled [13,34].

In our study with pharmacists and pharmacy technicians, we identified starting points to enhance and strengthen the knowledge of ultraviolet protection and sunscreen use. The majority of participants reported being aware of the importance of sun protection counseling, at least during the summer season. At the same time, the participants expressed their wish for further education on ultraviolet protection. There was a need for information provided by independent stakeholders, as most training and education opportunities currently seem to be offered by sunscreen manufacturers.

Based on our findings, potential topics for the education and training of pharmacists and pharmacy technicians are the application of sunscreen, the definition of skin type, and the practical application of skin type classification in counseling customers. Regarding the application of sunscreen, several aspects could be covered by the information provided by stakeholders in the area of health promotion and prevention. For instance, one issue is the amount of sunscreen that should be applied to the skin. In our study, several participants would recommend a (much) too small amount to customers. However, the knowledge of the estimated number of times an adult can apply sunscreen to the entire body by using a 200 ml bottle of sunscreen [24] was better in our sample compared to the general population [23]. It is conceivable that people who work in pharmacies are more familiar with package sizes and are, therefore, better able to estimate them. In particular, the three case studies used in our interviews showed that not all knew about the recommendation of health authorities regarding the time of application and re-application of sunscreen [26,27,28,29]. This finding opens up further opportunities for educational outreach among pharmacy personnel. Handouts that specifically address counseling situations in pharmacies may be helpful. In addition, webpages by dermatology associations such as the Canadian Dermatology Association may be helpful for pharmacy staff [35].

The classification proposed by Fitzpatrick [25] to define an individual’s skin type has gained worldwide acceptance. Sun protection measures can be derived based on skin types ranging from I to VI. Thus, knowledge and application of this classification can be helpful during the counseling of customers at pharmacies. To establish the use of skin type as a tool for advising customers on sun protection in pharmacies, it may be reasonable to focus on this in continuing education courses. The same applies to the so-called “UV index”, which is also recommended as a tool for determining appropriate sun protection measures [9].

For some participants, we found little knowledge of what sun protection factor means. The sun protection factor is defined as a measure of the amount of ultraviolet radiation required to produce sunburn. It seems to be more established than the term “UV index”; however, knowledge of the term sun protection factor seems to be necessary to advise customers adequately on the choice of sunscreen product. In a Malaysian study, about two-thirds of pharmacists were familiar with the definition of sun protection factor [31]. In the general population, about one-third was able to provide a correct definition [36,37]. Comparable to the recommendations on the application of sunscreen, a handout targeted at pharmacy personnel might be helpful. Since sunscreen is a seasonal product, according to the information provided by our participants, such a handout can help refresh the definition of sun protection factor in early summer.

The case studies included in our study underscore that pharmacies can play a major role in the prevention of skin cancer. About two-thirds of pharmacists in a previous study indicated that pharmacies are a good place for skin cancer prevention [30]. In our study, the three main reasons for buying sunscreen at the pharmacy were individual consultation, perceived higher product quality, and higher competency of the staff. However, our participants admitted to having little education in the field of skin cancer prevention. In addition, our case studies provided the first indication that a guideline for consultation could be useful, which maps the different aspects that need to be discussed. After all, sun protection means more than just sunscreen use, but this may not be present in the training courses of companies and manufacturers of sunscreen. In such training, product-specific information is in the foreground and the view for a holistic approach to sun protection may be missing. In addition, such a guide can be put to good use when routine sunscreen counseling is lacking or at the beginning of the summer.

### Strengths and Limitations

Our study aimed to explore the situation of the sale and counseling of sunscreens in pharmacies. Using different case studies helped pharmacists and pharmacy technicians to describe how they would act in this situation. This allowed us to obtain in-depth insights into different counseling situations, which would not be possible when using a standardized survey. Together with the participants, we could identify which pieces of information they might additionally need to advise their customers appropriately and which sources they would prefer for such information.

However, this study has some potential limitations that should be considered. Our study was limited to the Rhine-Neckar region (Southwestern Germany). Therefore, our findings may not be transferrable to the entirety of Germany. In the familiar interview setting, we took care to ensure that no assessment of the pharmacy employees’ level of knowledge was made and that they did not feel as if they were taking an exam. At the same time, this made it impossible to assess knowledge in a standardized manner; however, this was not the aim of this study.

## 5. Conclusions

Pharmacies can play an important role in promoting sun protection and selling sunscreens. Although they may be aware of their important role, our participants emphasized a lack of knowledge. It would be useful to further investigate the level of knowledge about sun protection among pharmacy staff in large representative surveys in Germany and in other countries. This will allow comparisons to be made between countries and will help to develop support services that are as tailor-made as possible. In Germany, assistance in the form of counseling guidelines, handouts targeted for pharmacies, and education by stakeholders in the field of skin cancer prevention may help pharmacists and pharmacy technicians provide their customers with evidence-based counseling and education on sun protection behavior. Including pharmacy personnel as key players in the field of skin cancer prevention opens new possibilities to fight the increasing incidence of skin cancer.

## Figures and Tables

**Table 1:** Verbatim responses of pharmacists and pharmacy technicians who would recommend using too little sunscreen.

ID	Verbatim Responses
P02	“25 milliliters per time, I would estimate.”
P04	“For children, so one tablespoon, for adults rather two.”
P05	“A lot of people don’t know that a little bit is actually always enough, so you don’t have to cover your whole face with sunscreen. So, I always recommend using it like a day cream. Just apply it like a day cream to your face and that is actually enough.”

**Table 2:** Examples of verbatim responses expressing the wish for independent information on sun protection detached from manufacturing companies.

ID	Verbatim Responses
P03	“Yeah, from the associations wouldn’t be bad, so from, from a more neutral, body.”
P08	“Something independent would be nice.”
P18	“[…] perhaps from a dermatological association, because as I said, a part, a large part of the information is of course industry-driven, which is suboptimal.”
P19	“So if I were to wish for information, I would only want independent, […] not company-dependent [information]. Although those can be good, of course, but I have noticed that as soon as there’s company-dependent information, one thing or another may be correct, but you’re always being trained towards that one product. And I don’t want to be trained on the product, I want to be trained on the ingredients, on the pros and cons of the substances, and the disadvantages of the substances. And that’s why I need a neutral body, that would be the State Chamber of Pharmacists, I would have full confidence in that. I also have confidence in the articles that are in the Pharmacy Newspaper, but if it came from medical circles, I would have nothing against it, so from dermatologists or whatever. A desire for more information—yes, but it would have to be scientific and independent.”

**Table 3:** Content of counseling during case study I.

	Participant Number	01	02	03	04	05	06	07	08	09	10	11	12	13	14	15	16	17	18	19	20	Sum
Content																						
Consideration of children’s age																						12
Consideration of children’s and adults’ skin type																						9
Consideration of special skin needs (e.g., sensitive skin or skin diseases)																						12
Recommendation of sunscreen with a high sun protection factor																						13
Recommendation of water-resistant sunscreen																						7
Recommendation of using a sufficient amount of sunscreen																						7
Recommendation of regular reapplication of sunscreen																						8
Recommendation of wearing (ultraviolet-protective) clothes																						8
Recommendation of wearing a hat																						5
Recommendation of staying in the shade																						5
Sum of aspects		2	7	6	1	3	4	4	2	2	7	4	6	5	5	2	2	4	7	8	5	

Cells with gray background mean that this aspect was addressed during counseling.

## Data Availability

The data presented in this study are available on request from the corresponding author.
